# Improved adherence to Mediterranean Diet in adults with type 1 diabetes mellitus

**DOI:** 10.1007/s00394-018-1777-z

**Published:** 2018-07-17

**Authors:** Minerva Granado-Casas, Nuria Alcubierre, Mariona Martín, Jordi Real, Anna M. Ramírez-Morros, Maribel Cuadrado, Núria Alonso, Mireia Falguera, Marta Hernández, Eva Aguilera, Albert Lecube, Esmeralda Castelblanco, Manel Puig-Domingo, Dídac Mauricio

**Affiliations:** 1 Department of Endocrinology and Nutrition, Health Sciences Research Institute and University Hospital Germans Trias i Pujol, 08916 Badalona, Spain; 2 Epidemiology and Public Health, International University of Catalonia, Barcelona, Spain; 3 Unit Support of Research, Institut d’Investigació en Atenció Primària Jordi Gol (IDIAP Jordi Gol), Barcelona, Spain; 4 Department of Endocrinology and Nutrition, University Hospital Germans Trias i Pujol, Badalona, Spain; 5 Department of Endocrinology and Nutrition, University Hospital Arnau de Vilanova, Lleida, Spain; 6 Primary Health Care Centre Igualada Nord, Consorci Sanitari de l’Anoia, Igualada, Spain; 7 Biomedical Research Institute, University of Lleida, Lleida, Spain; 8 Centre for Biomedical Research on Diabetes and Associated Metabolic Diseases (CIBERDEM), Instituto de Salud Carlos III, Barcelona, Spain

**Keywords:** Type 1 diabetes mellitus, Mediterranean Diet, Dietary habits, Food intake, Intake pattern, Medical nutrition therapy

## Abstract

**Purpose:**

We aimed to assess food intake and adherence to the Mediterranean Diet in patients with T1D compared with nondiabetic individuals.

**Methods:**

This was an observational, multicenter study in 262 T1D subjects and 254 age- and sex-matched nondiabetic subjects. A validated food-frequency questionnaire was administered. The alternate Mediterranean Diet Score (aMED) and alternate Healthy Eating Index (aHEI) were assessed. The clinical variables were also collected. The analysis of data included comparisons between groups and multivariate models.

**Results:**

Compared to the controls, the patients with T1D had a higher intake of dairy products (*p* < 0.001), processed meat (*p* = 0.001), fatty fish (*p* = 0.009), fruits and vegetables (*p* < 0.001), nuts (*p* = 0.011), legumes (*p* < 0.001), potatoes (*p* = 0.045), and bread (*p* = 0.045), and a lower intake of seafood (*p* = 0.011), sweets (*p* < 0.001), and alcohol drinks (*p* = 0.025). This intake pattern resulted in a higher consumption of complex carbohydrates (*p* = 0.049), fiber (*p* < 0.001), protein (*p* < 0.001), polyunsaturated fatty acids (PUFA) (*p* = 0.007), antioxidants (*p* < 0.001), vitamins (*p* < 0.001), and minerals (*p* < 0.001). The frequency of patients with T1D and low aMED score (23.2%) was lower than that of the controls (35.4%; *p* = 0.019). The overall multivariate analysis showed that, among other factors, being a T1D subject was associated with improved aMED and aHEI scores (*p* = 0.006 and *p* < 0.001). In patients with T1D, residing in a nonurban area was associated with improved aMED and aHEI scores (*p* = 0.001 and *p* < 0.001).

**Conclusions:**

Adult patients with T1D showed healthier dietary habits and a higher adherence to the Mediterranean Diet than nondiabetic subjects. Residing in a nonurban area is associated with an improved dietary pattern.

**Electronic supplementary material:**

The online version of this article (10.1007/s00394-018-1777-z) contains supplementary material, which is available to authorized users.

## Introduction

Medical nutrition therapy (MNT) and physical activity, in addition to insulin therapy, are cornerstones in the management of type 1 diabetes (T1D). MNT produces health benefits on glucose control, lipid profile, weight management, and maintenance of muscle mass [1–3]. Different scientific societies have issued nutritional recommendations for the medical nutrition management of T1D [2–5]. MNT is also an integral component of diabetes self-management education [2]. The handling of dietary carbohydrate content and insulin management are mainly targeted to enable the patients with T1D to self-manage the disease [2, 3]; furthermore, healthy eating recommendations are usually included in the MNT, because these patients are regarded as a high cardiovascular risk group [2, 3, 5].

In our region, the dietary habits are identified by the Mediterranean Diet (MedDiet). This dietary pattern includes moderate intake of energy, low intake of animal fat, high intake of fruits, vegetables, whole grains, legumes, olive oil and moderate intake of fish, poultry, and red wine, together with regular physical activity (25–30 min every day) [3, 6, 7]. A number of epidemiological and intervention studies have demonstrated the potential benefits of the MedDiet in preventing cardiovascular diseases [6–10]. The MedDiet has been associated with a reduction of 9% overall mortality from cardiovascular diseases and all causes [7, 10].

Several studies in T1D have assessed the comparison between the dietary habits and the American Diabetes Association (ADA) or the European Association for the Study of Diabetes (EASD) nutritional recommendations [11–15]. Higher intakes of total fat and saturated fatty acids (SFA) have been shown in European patients with T1D [11–14]. Furthermore, the intake of carbohydrates and fiber tends to be low in this population [11, 14, 15], although the protein consumption is adequate according to the established guidelines [12, 13]. The intake of micronutrients is high in children, adolescents, and adults with T1D [11, 15]. The EURODIAB Study reported a positive relationship between a higher intake of fat and SFA, and a low intake of carbohydrates and fiber with glycemic control and cardiovascular risk factors [16, 17]. Most studies have been only performed in patients with T1D, and there are very few studies that compare the dietary habits between patients with T1D and a nondiabetic group [18–20]. In addition, to the best of our knowledge, there are only two studies that assessed the adherence to the MedDiet and healthy eating pattern in a small group of Canadian patients with T1D, but without a control group [21, 22]. Furthermore, there are only two studies with a case–control design that determined the dietary habits in children and adolescents with T1D [23, 24].

Because patients with T1D receive regular nutritional education, we hypothesized that they would have healthier eating habits than nondiabetic subjects. In a Mediterranean country like ours, MNT includes recommendations for adhering to a MedDiet pattern [4]. However, there are no data on the impact of real-world MNT practices on the dietary pattern of subjects with T1D in comparison with their nondiabetic counterparts. Thus, the aim of this study was to assess the dietary habits and adherence to the MedDiet in patients with T1D and their comparison to nondiabetic counterparts. In addition, we aimed to investigate the factors related to the MedDiet and food intake pattern.

## Materials and methods

### Subjects

This was an observational and two-center study. A sample of 513 participants was recruited: 259 patients with T1D and 254 nondiabetic subjects matched for age and sex in each location [healthcare areas of Lleida (center 1) and Barcelona (center 2)] between January 2013 and May 2015. The location of Lleida is a mixed rural and semi-urban area, and Barcelona is a fully urban area. The cases were patients diagnosed with T1D who were regularly cared for at their reference hospital (University Hospital Arnau of Vilanova and University Hospital Germans Trias i Pujol). Participation in the study was offered to outpatients with T1D in both departments until the study sample was complete. The inclusion criteria of cases were as follows: diagnosis of T1D with a duration of more than 1 year; age > 18 years for both cases and control participants. The exclusion criteria for both groups were as follows: being a healthcare professional, participants who showed physical and cognitive deterioration as dementia, mental diseases, and known cardiovascular diseases (ischemic heart disease, cerebrovascular disease, peripheral arterial disease, and heart failure) or previous diabetic foot disease, pregnancy, and renal insufficiency (estimated glomerular filtration rate < 60 mL/min). Furthermore, for T1D, we excluded patients with a condition that requires additional MNT measures, i.e., macroalbuminuria (urine albumin/creatinine ratio > 299 mg/g). The control participants were also excluded if they had an HbA1c value ≥ 6.5% [25].

### Study design

In center 1, from an initial sample of 170 patients with T1D who were contacted to participate in the study, 128 subjects accepted to participate, and 3 were excluded because of the exclusion criteria, resulting in a target sample of 125 (Supplemental Fig. 1). However, 3 additional subjects were excluded after their inclusion because of the identification of exclusion criteria, resulting in a final sample of 122 cases. The corresponding control group was identified from a population-based study, which was performed simultaneously in that center [25]. The nondiabetic subjects were randomly selected with matching for age (in strata of 5 years) and sex with cases, resulting in a final sample of 125 controls.

In center 2, a total of 160 patients with T1D were contacted; 148 agreed to participate. From this group, 11 were excluded, because they had met the exclusion criteria. The final sample was 137 patients with T1D. The control group was recruited at the same time as the cases; from a sample of 160 controls, 151 accepted and 22 were excluded because of the exclusion criteria, resulting in a final sample of 129 controls (Supplemental Fig. 1).

The study was approved by the local Ethics Committee of both centers, and written informed consent was obtained from all the participants.

### Clinical variables

The clinical variables collected for each study group are shown in Table 1. Blood and urine samples were collected, and the biochemical variables were determined using the standard methods. Cardiovascular disease was excluded based on detailed anamnesis and careful review of all clinical records. The use of any medication, including any antihypertensive or lipid-lowering agents, was also recorded. Physical activity was assessed using the validated method of Bernstein et al. [26] and Cabrera de León et al. [27]. Physical activity was classified as either regular physical activity if the subject conducted any type of physical activity that requires 4 METS (The Metabolic Equivalent) minimum, such as walking or cycling, for more than 25 min/day or sedentary if the subject spent less than 25 min/day in physical activity.

**Table 1:** Clinical and demographic characteristics of the study group

Characteristics	T1D (*n* = 259)	Control (*n* = 254)	*p* ^a^
Age (years)	43.7 ± 11.2	45.4 ± 11.2	0.10
Men (sex)	118 (45.6)	122 (48.0)	0.64
Educational level			0.05
Less than primary	15 (6.0)	6 (2.4)	
Primary	74 (29.8)	56 (22.2)	
Secondary	97 (39.1)	116 (46.0)	
Graduate or higher	62 (25.0)	74 (29.4)	
Smoking			0.43
No	130 (50.2)	112 (44.1)	
Yes	70 (27.0)	78 (30.7)	
Former smoker	59 (22.8)	64 (25.2)	
Regular physical activity	186 (71.8)	130 (51.2)	< 0.001
Diabetes duration (years)	21.5 ± 10.5	–	–
BMI (kg/m^2^)	25.6 ± 4.1	25.9 ± 4.0	0.44
Waist circumference (cm)	88.3 ± 12.7	92.5 ± 11.8	< 0.001
Systolic blood pressure (mmHg)	126.6 ± 17.4	122.6 ± 15.5	0.009
Diastolic blood pressure (mmHg)	73.7 ± 9.5	77.3 ± 9.4	< 0.001
Antihypertensive treatment	64 (24.7)	18 (7.1)	< 0.001
Dyslipidemia treatment	102 (39.4)	39 (15.4)	< 0.001
HbA1c (%)	7.6 ± 0.9	5.5 ± 0.4	< 0.001
HbA1c (mmol/mol)	59.4 ± 10.4	37.0 ± 4.1	< 0.001
Total cholesterol (mg/dL)	180.6 ± 28.5	194.4 ± 37.9	< 0.001
HDLc (mg/dL)	64.7 ± 16.0	61.4 ± 16.0	0.025
LDLc (mg/dL)	101.9 ± 23.4	115.9 ± 33.5	< 0.001
Triglycerides (mg/dL)	73.7 ± 37.8	94.3 ± 53.2	< 0.001

Values are mean ± SD or *n* (%)

*BMI* body mass index, *HbA1c* glycated hemoglobin, *HDLc* high-density lipoprotein cholesterol, *LDLc* low-density lipoprotein cholesterol, *T1D* type 1 diabetes

^a^*p* was calculated according to the method of Benjamini and Hochberg

### Assessment of food pattern intake and adherence to dietary index

The Food-Frequency Questionnaire Consumption (FFQC) was administered by personal interview by specialized and trained researchers. This is a semiquantitative questionnaire, validated and adapted for the Spanish population and based on the Nurses’ Health Study [28, 29]. The questionnaire contains 101 items that ask each individual about his or her usual consumption over the previous year’s visit. To assess the degree of adherence to the MedDiet, we used the alternate Mediterranean Diet Score (aMED) based on the Mediterranean Diet scale [30]. We also determined the alternate Healthy Eating Index (aHEI) based on the Dietary Guidelines for Americans and the Food Guide Pyramid [31]. The aMED includes vegetables, legumes, fruits, nuts, grains (only whole grain), red and processed meat, fish, monounsaturated fatty acids (MUFA)-to-SFA ratio, and alcohol intake. The score ranges from 0 (minimal adherence) to 9 (maximal adherence). The aHEI includes vegetables, fruit, nuts and soy, white and red meats, cereal fiber, trans fat, polyunsaturated and saturated fat, and alcohol intake. Energy and nutrient intake was obtained according to standardized measurement units. Daily nutrient intake was calculated adjusting for energy intake and expressed as units/day according to the standardized methods for the FFQC.

### Sample size

The sample size was calculated to detect the differences between the study groups based on previous results from a pilot study performed to assess the potential output (60 cases and 60 controls) in which the mean and standard deviation values of aMED were estimated in both groups: T1D 4.2 ± 1.4 and controls 3.7 ± 1.4. Based on this difference, using a significance level of 5% (*α* = 0.05) and a statistical power of 80% (*β* = 0.2), a sample of 125 cases and 125 controls was necessary to detect a statistically significant difference in the aMED score. Finally, based on the availability of a sufficient number of potential participants, we aimed to conduct the study in two different geographical areas, which also allowed us to increase the statistical power of the study.

### Data analyses

An initial descriptive comparison between groups of all variables was performed. The quantitative variables were summarized using the mean and standard deviation (SD) values, and the qualitative variables were summarized using the absolute (*n*) and relative frequencies (%). Statistical significance was assessed using the Chi-squared test to assess the differences in the frequencies. The mean of clinical determinations, daily food and nutrient intake, and the dietary quality index were compared using Student’s *t* test. The “BH” (aka “fdr”) and “BY” methods of Benjamini, Hochberg, and Yekutieli were performed; they control the false discovery rate and the expected proportion of false discoveries among the rejected hypotheses. The false discovery rate is a less stringent condition than the family-wise error rate, so these methods are more powerful than the others. The multivariate regression models were developed to analyze the relationship between group (case and control) and dietary quality index adjusted by the potential confounders. The models were adjusted by variables that were statistically significant in the bivariate analysis or were clinically associated with diabetes. The conditional logistic regression models were designed to explain low aMED adherence (aMED low: 0–2) and adjusting linear regression models were performed for aHEI. The goodness-of-fit assumption using the Hosmer–Lemeshow test for logistic models was assessed, and for the linear regression model, the Kolmogorov–Smirnov test was performed. The estimate measures were the odds ratio (OR) with 95% confidence interval (95% CI) and with logistic regression models, and the effect (*β*) and standard error (± SE) with linear regression. In all the tests, a *p* < 0.05 was considered to be statistically significant.

## Results

The clinical and demographic characteristics of the study groups are shown in Table 1. The patients with T1D showed lower waist circumference (*p* < 0.001) than the control group. However, the patients with T1D performed regular physical activity more frequently (*p* < 0.001), and had a high frequency of treatment for hypertension (*p* < 0.001) and dyslipidemia (*p* < 0.001). Furthermore, the patients with T1D showed a better lipid profile.

### Dietary habits

The patients with T1D had a healthier food intake pattern than the control group, as shown in Table 2. Compared to the controls, the subjects with T1D had a higher intake of dairy products (*p* < 0.001), processed meat (*p* = 0.001), fatty fish (*p* = 0.009), fruits and vegetables (*p* < 0.001), nuts (*p* = 0.011), legumes (*p* < 0.001), potatoes (*p* = 0.045) and bread (*p* = 0.045), and a lower intake of seafood (*p* = 0.011), sweets (*p* < 0.001) and alcohol drinks (*p* = 0.025).

**Table 2:** Dietary quality index and daily food intake of the study groups

Items	T1D (*n* = 259)	Control (*n* = 254)	*p* ^a^
Dietary quality index			
aMED	3.7 ± 1.6	3.2 ± 1.8	0.009
aMED			0.019
Low (0–2)	60 (23.2)	90 (35.4)	
Moderate (3–5)	167 (64.5)	139 (54.7)	
High (6–9)	32 (12.4)	25 (9.8)	
aHEI	40.7 ± 6.5	37.6 ± 6.2	< 0.001
aHEI			0.011
Low (< 45)	196 (75.7)	218 (85.8)	
High (≥ 45)	63 (24.3)	36 (14.2)	
Daily food intake (g/day)			
Dairy products	401.6 ± 240.8	309.6 ± 195.0	< 0.001
Eggs	20.3 ± 11.9	20.6 ± 11.0	0.82
White meat	36.1 ± 22.0	35.9 ± 21.1	0.93
Red meat	51.9 ± 38.7	55.7 ± 37.9	0.35
Processed meat	43.6 ± 32.2	34.5 ± 25.3	0.001
Meat	129.1 ± 55.1	125.0 ± 52.7	0.48
Lean fish	31.4 ± 36.5	30.2 ± 21.2	0.72
Fatty fish	36.2 ± 29.3	29.2 ± 23.2	0.009
Seafood	9.6 ± 8.5	12.3 ± 12.2	0.011
Fish	77.5 ± 48.9	70.6 ± 37.8	0.13
Fruits and vegetables	490.1 ± 233.9	372.0 ± 163.4	< 0.001
Nuts	14.7 ± 29.3	9.0 ± 14.0	0.011
Legumes	34.2 ± 25.1	25.9 ± 19.7	< 0.001
Cereals and pasta	82.8 ± 44.5	77.7 ± 42.6	0.29
Potatoes	55.7 ± 39.6	48.1 ± 37.4	0.045
Bread	104.5 ± 52.6	93.8 ± 56.1	0.045
Sweets	17.4 ± 20.9	38.5 ± 35.1	< 0.001
Vegetable fats	40.0 ± 16.7	38.8 ± 19.6	0.52
Animal fats	0.2 ± 1.0	0.2 ± 0.7	0.72
Alcohol drinks	92.5 ± 168.8	139.0 ± 244.5	0.025
Non-alcoholic beverages	1504.1 ± 549.8	1447.8 ± 467.4	0.29
Coffee and tea	421.7 ± 280.4	448.0 ± 312.3	0.40
Prepared meals	69.2 ± 83.8	61.3 ± 54.7	0.29
Salt	1.1 ± 1.8	0.9 ± 1.1	0.19

Values are mean ± SD or *n* (%)

*T1D* type 1 diabetes, *aMED* alternate Mediterranean Diet index, *aHEI* alternate Healthy Eating Index

^a^*p* was calculated according to the method of Benjamini and Hochberg

Consistent with this food pattern, there was also a differential intake of nutrients (Supplemental Table 1). The patients with T1D showed a higher intake of complex carbohydrates (*p* = 0.049), soluble and insoluble fiber (*p* < 0.001), protein (*p* < 0.001), and PUFA (*p* = 0.007); the latter included intake of omega 3 (*p* < 0.001), omega 6 (*p* = 0.016), linoleic acid (*p* = 0.016), α-linolenic acid (*p* < 0.001), eicosapentaenoic acid (EPA) (*p* = 0.049), and docosahexaenoic acid (DHA) (*p* = 0.016). Furthermore, subjects with T1D also showed a higher consumption of all vitamins (*p* < 0.001; except vitamin B12), carotenoids (*p* < 0.001), and minerals (*p* < 0.001; except for iron intake). In contrast, the control group showed a higher intake of energy (*p* = 0.011), sugar (*p* < 0.001), stearic acid (*p* = 0.021), and alcohol (*p* = 0.002); they also had a higher dietary glycemic index (*p* = 0.002).

### Alternate Mediterranean Diet Score

**Fig. 1:**
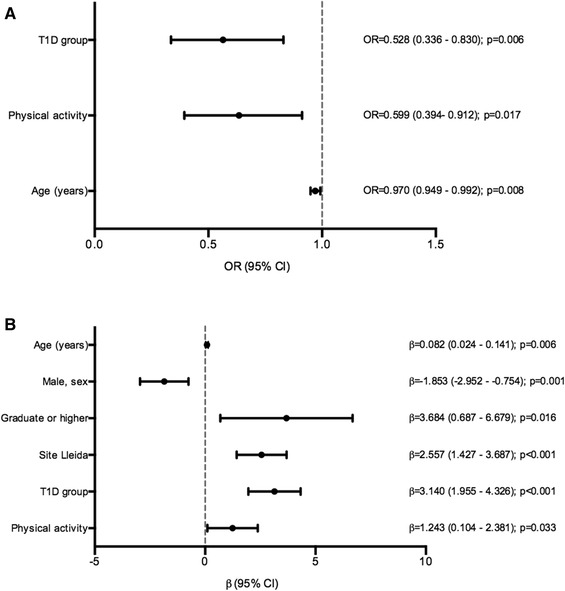
Multivariate analysis for the alternate Mediterranean Diet Score (aMED) and alternate Healthy Eating Index (aHEI) of the study groups. **a** Multivariate logistic regression for the aMED low group (0–2 points). Hosmer–Lemeshow test *p* value: 0.08. **b** Multivariate linear regression for the aHEI. Multiple *R*^2^: 0.16; adjusted *R*^2^: 0.14. T1D, type 1 diabetes

The dietary quality index of each of the study groups is shown in Table 2. Compared to the control group, the participants with T1D showed a higher aMED score (*p* = 0.009). Furthermore, the frequency of patients with T1D with poor adherence to a MedDiet (low aMED score) was lower (*p* = 0.019). However, both groups had a moderate mean aMED score (3.7 ± 1.6, T1D; 3.2 ± 1.8, control). The multivariate logistic analysis showed that being a subject with T1D (*p* = 0.006), increased physical activity (*p* = 0.017) and increasing age (*p* = 0.008) were negatively related to a low aMED score; thus, all these variables were associated with a higher adherence to a MedDiet (Fig. 1a, Supplemental Table 2).

### Alternate Healthy Eating Index

Patients with T1D also showed a higher aHEI score than the controls (*p* < 0.001) (Table 2). In addition, consistent with the results of aMED score, the frequency of patients with T1D and with a low aHEI score (defined as aHEI < 45 points) was lower (75.7%, T1D vs. 85.8%, controls; *p* = 0.011). Moreover, the mean of aHEI was relatively low in both groups (40.7 ± 6.5, T1D; 37.6 ± 6.2, controls). The multivariate linear analysis revealed that the factors associated with higher aHEI scores were the T1D group (*p* < 0.001), high educational level (*p* = 0.016), residing in the region of Lleida (*p* < 0.001), increased physical activity (*p* = 0.033), and increasing age (*p* = 0.006) (Fig. 1b, Supplemental Table 2). However, the male sex showed a negative association with this index (*p* = 0.001).

### Factors associated with the dietary pattern in T1D

**Fig. 2:**
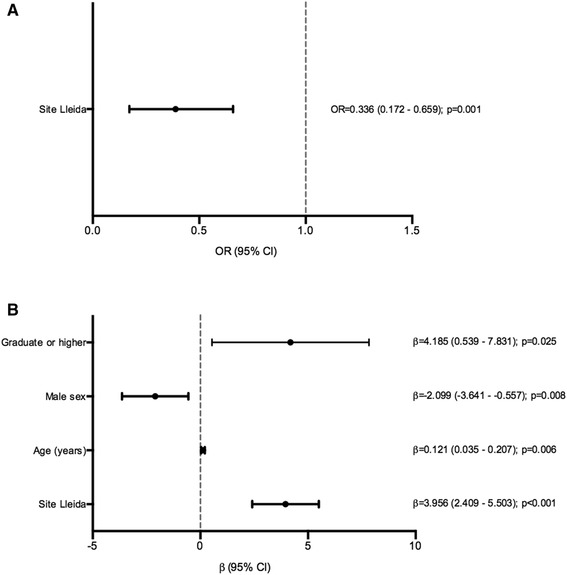
Multivariate analysis for the alternate Mediterranean Diet Score (aMED) and alternate Healthy Eating Index (aHEI) of the type 1 diabetes group. **a** Multivariate logistic regression for the aMED low group (0–2 points). Hosmer–Lemeshow test *p* value: 0.163. **b** Multivariate linear regression for the aHEI. Multiple *R*^2^: 0.20; adjusted *R*^2^: 0.15

The multivariate analyses within the T1D group showed that the factor related to a high adherence to the MedDiet was residence in the region of Lleida (rural/semi-urban area) (*p* = 0.001) (Fig. 2a, Supplemental Table 3). The factors related to aHEI score were residence in Lleida (*p* < 0.001), high educational level (*p* = 0.025), and age (*p* = 0.006). The male sex was negatively associated with aHEI (*p* = 0.008) (Fig. 2b, Supplemental Table 3).

## Discussion

In this study, we observed healthier dietary habits in patients with T1D in comparison with their nondiabetic counterparts. This finding resulted in a higher adherence to the MedDiet and healthier eating patterns, with higher aMED and aHEI scores in patients with T1D. In addition, we found a positive relationship between T1D, physical activity, and age with both dietary scores. The participants who were residing in the rural/semi-urban area and had a high educational level showed an increased aHEI score, whereas the male sex was negatively related to it. This is the first study that assesses food and nutrient intake, adherence to the MedDiet, and a healthy eating pattern in adult patients with T1D. Moreover, this study was specifically designed to address this question. To the best of our knowledge, no study has addressed the issue of the adherence to the MedDiet between adult subjects with T1D and a nondiabetic group.

Consistent with the previous findings [11–15], despite patients with T1D showing a moderate aMED and healthier dietary pattern, the adherence to the nutritional recommendations cannot be regarded as optimal in terms of the recommendations [2–5]. There are only three studies that compared the dietary habits between patients with T1D and nondiabetic subjects [18–20]. Snell-Bergeon et al. [18] and Jaacks et al. [19] observed a lower energy intake and higher intakes of protein and vegetables in patients with T1D; this observation is concordant with the current findings. However, they showed a low intake of carbohydrates, high intake of fat derived from SFAs, and similar glycemic index, which is in contrast with our results. In addition, an audit of dietary management in adults with T1D found no differences in terms of macronutrient intake between the patients with T1D and a control group, although there was a potential selection bias, and both groups were not matched for age and sex [20]. Actually, there are only two studies using a case–control design that showed healthier dietary habits in children and adolescents with T1D [23, 24]. However, it is conceivable that the dietary patterns of children and adolescents with T1D are mainly determined by the parental dietary habits [23, 24]. Therefore, ours is the first study showing healthier dietary patterns in adult patients with T1D and a matched nondiabetic group.

In the current study, patients with T1D had more frequently higher aMED and aHEI scores in comparison with a control group. Nevertheless, the mean of aHEI was low in both groups according to the index classification. To date, there is no study that has assessed the adherence to the MedDiet and healthy eating using this study design. Only a Canadian study has shown a moderate adherence to the MedDiet with less than half of the patients with T1D (49%) showing a high Canadian Healthy Eating Index (C-HEI); this finding is similar to our results [21, 22]. However, that study was performed using a small sample without a nondiabetic control group.

We must underscore that the fact of not residing in an urban area was the factor that was closely and consistently associated with improved dietary quality indexes in patients with T1D. This points to a relevant influence of the socio-geographical context in determining the dietary pattern of any given subject. In addition, discordant results found by different studies in T1D may, in part, be attributed to this factor. This is also an important finding for future studies, as the control group should be carefully selected.

In our study, the subjects with a high educational level, regular physical activity, and older age showed an improved healthy lifestyle behavior independent of the disease duration. It is reasonable to think that a healthy lifestyle is more likely to be adopted in older patients and that it is maintained over time [32]. In addition, as in other studies, men show an unhealthier dietary pattern than women [22, 33]. We did not find a relationship between aMED and aHEI with the presence of dyslipidemia or hypertension, although an inverse association between lipid profile, blood pressure, and a healthy lifestyle has been reported in patients with T1D [22]. Therefore, in our population, dyslipidemia and hypertension do not seem to be potential factors that enhance the adherence to a healthier dietary pattern.

The current study has several limitations. No relationship of direct causality can be established between the different variables associated with the MedDiet, because the changes in lifestyle habits produced over time cannot be addressed with a study like the current one. Although the current results point to the probable influence on the better dietary habits of T1D patients of the educational dietary intervention (usually mainly focused on carbohydrate counting) that they receive from the treating healthcare professionals, we cannot conclude that this is the case. Unfortunately, we did not assess the knowledge of patients about the dietary management of diabetes. This study has several strengths. The large number of participants, the multicenter design, and a well-characterized sample allow us to establish the variability of different populations and lifestyles associated with them. Furthermore, we could study the different pattern in two different areas of the same region, North-Eastern Spain, that differed by their urban and rural/semi-urban locations. Moreover, this is the first study that assessed the adherence to the MedDiet between adults with T1D and a nondiabetic control group.

In conclusion, in our Mediterranean region, adults with T1D showed a healthier food intake pattern and, specifically, an improved adherence to the MedDiet than their nondiabetic counterparts. However, additional research is warranted in this field and in the identification of the educational strategies that need to be adopted to enhance the adherence of patients with T1D to a healthy diet.

## Electronic supplementary material

Below is the link to the electronic supplementary material.

Supplementary material 1 (DOCX 36 KB)

Supplementary material 2 (DOCX 26 KB)

Supplementary material 3 (DOCX 18 KB)

Supplementary material 4 (DOCX 17 KB)
