# Supplementary material for: Improved adherence to Mediterranean Diet in adults with type 1 diabetes mellitus
Source: Eur J Nutr. 2018 Jul 17;58(6):2271–9. doi: 10.1007/s00394-018-1777-z (PMC6689285; doi:10.1007/s00394-018-1777-z)
Supplement: Supplementary file 1 — Supplementary material 1 (DOCX 36 KB) [file 394_2018_1777_MOESM1_ESM.docx]

**TYPE 1 DIABETES GROUP**

**CENTER 1**

**Total contacted patients**

**n = 170**

Accepted to participate

**n = 128**

Included

**n = 125**

-Previous cardiovascular disease

-Pregnancy

-Glomerular filtration rate < 60 ml/min

**n = 3 excluded**

Final sample

**n = 122**

Previous cardiovascular disease

**n = 3 excluded**

**CENTER 2**

**Total contacted patients**

**n = 160**

Accepted to participate

**n = 148**

Final sample

**n = 137**

-Glomerular filtration rate < 60 ml/min

-Macroalbuminuria

-Previous cardiovascular disease

**n = 11 excluded**

**CONTROL GROUP**

**CENTER 1**

**Controls of a previous published study**

**n = 594**

Selection and matching for age and sex with cases

Final sample

**n = 125**

**CENTER 2**

**Total contacted controls**

**n = 160**

Accepted to participate

**n = 151**

Final sample

**n = 129**

-Non-adult

-HbA1c value ≥ 6.5 %

-without clinical or

nutritional data

**n = 22 excluded**

Supplemental Fig. 1 Flow chart of study subject recruitment.
