# Supplementary material for: Improved adherence to Mediterranean Diet in adults with type 1 diabetes mellitus
Source: Eur J Nutr. 2018 Jul 17;58(6):2271–9. doi: 10.1007/s00394-018-1777-z (PMC6689285; doi:10.1007/s00394-018-1777-z)
Supplement: Supplementary file 2 — Supplementary material 2 (DOCX 26 KB) [file 394_2018_1777_MOESM2_ESM.docx]

Supplemental Table 1. Daily nutrient intake of the study groups^a^

| **Nutrients intake (units/day)^b^** | **T1D**  **(n=259)** | **Control**  **(n=254)** | **p^c^** |
| --- | --- | --- | --- |
| Energy intake (Kcal) | 2,074.8 ± 513.2 | 2,212.2 ± 622.1 | 0.011 |
| Glycemic index (%) | 83.9 ± 17.1 | 89.1 ± 19.7 | 0.002 |
| Carbohydrate (g) | 192.3 ± 33.9 | 194.9 ± 34.1 | 0.46 |
| % of energy intake | 37.1 ± 6.5 | 37.6 ± 6.6 | 0.40 |
| Complex carbohydrate (g) | 88.4 ± 19.2 | 84.7 ± 19.8 | 0.049 |
| Sugar (g) | 80.8 ± 23.5 | 108.6 ± 40.4 | <0.001 |
| Fiber (g) | 23.0 ± 6.0 | 19.8 ± 4.9 | <0.001 |
| Soluble fiber (g) | 3.6 ± 1.2 | 2.9 ± 0.8 | <0.001 |
| Insoluble fiber (g) | 13.3 ± 4.2 | 11.1 ± 3.9 | <0.001 |
| Protein (g) | 97.6 ± 14.8 | 92.1 ± 14.4 | <0.001 |
| % of energy intake | 18.9 ± 2.9 | 17.7 ± 3.0 | <0.001 |
| Total fat (g) | 102.5 ± 15.3 | 100.1 ± 16.6 | 0.11 |
| % of energy intake | 44.4 ± 6.6 | 43.5 ± 7.2 | 0.16 |
| SFA (g) | 26.3 ± 4.8 | 26.5 ± 4.9 | 0.69 |
| MUFA (g) | 52.2 ± 10.6 | 50.8 ± 12.4 | 0.21 |
| PUFA (g) | 17.3 ± 5.1 | 16.1 ± 3.7 | 0.007 |
| Omega 3 (g) | 1.7 ± 0.4 | 1.5 ± 0.4 | <0.001 |
| Omega 6 (g) | 15.5 ± 5.1 | 14.5 ± 3.6 | 0.016 |
| Trans fat (g) | 1.0 ± 0.5 | 1.0 ± 0.4 | 0.96 |
| Cholesterol (mg) | 287.2 ± 70.5 | 290.2 ± 72.1 | 0.70 |
| Palmitic acid (16:0) (g) | 16.1 ± 2.5 | 16.0 ± 2.4 | 0.70 |
| Stearic acid (18:0) (g) | 6.0 ± 1.1 | 6.3 ± 1.5 | 0.021 |
| Oleic acid (18:1ω-9) (g) | 49.6 ± 10.4 | 48.2 ± 12.2 | 0.21 |
| Linoleic acid (18:2ω-9) (g) | 15.4 ± 5.1 | 14.3 ± 3.6 | 0.016 |
| α-linolenic acid (18:3ω-9) (g) | 1.2 ± 0.2 | 1.1 ± 0.2 | <0.001 |
| Arachidonic acid (20:4ω-6) (g) | 0.2 ± 0.0 | 0.3 ± 0.0 | 0.84 |
| EPA (20:5ω-3) (g) | 0.2 ± 0.1 | 0.1 ± 0.1 | 0.049 |
| DHA (22:6ω-3) (g) | 0.3 ± 0.2 | 0.2 ± 0.2 | 0.016 |
| Alcohol (g) | 6.1 ± 10.3 | 10.1 ± 16.3 | 0.002 |
| Caffeine (g) | 258.4 ± 217.1 | 253.7 ± 216.8 | 0.84 |
| Water (g) | 2,905.8 ± 687.5 | 2,861.2 ± 604.1 | 0.50 |
| Vitamin A (µg) | 1,242.9 ± 676.4 | 942.4 ± 415.3 | <0.001 |
| Retinol (µg) | 349.7 ± 408.5 | 336.6 ± 296.5 | 0.73 |
| Carotene (µg) | 868.4 ± 493.0 | 605.1 ± 325.6 | <0.001 |
| α carotene (µg) | 647.9 ± 558.1 | 436.8 ± 379.0 | <0.001 |
| β carotene (µg) | 4,712.5 ± 2,652.5 | 3,281.6 ± 1,746.3 | <0.001 |
| β cryptoxanthin (µg) | 319.8 ± 204.6 | 239.1 ± 136.5 | <0.001 |
| Lutein+zeoxanthin (µg) | 4,059.1 ± 3,142.8 | 2,628.6 ± 1,626.0 | <0.001 |
| Lycopene (µg) | 4,430.8 ± 2,078.0 | 4,132.4 ± 2,225.3 | 0.15 |
| Folate (µg) | 288.4 ± 77.1 | 247.8 ± 60.4 | <0.001 |
| Vitamin B_12_ (mg) | 8.4 ± 3.6 | 8.3 ± 3.1 | 0.88 |
| Vitamin B_6_ (mg) | 2.0 ± 0.5 | 1.8 ± 0.5 | 0.013 |
| Vitamin C (mg) | 115.6 ± 59.0 | 92.5 ± 43.0 | <0.001 |
| Vitamin D (mg) | 4.3 ± 1.7 | 3.9 ± 1.4 | 0.001 |
| Vitamin E (mg) | 14.6 ± 3.4 | 13.4 ± 3.4 | <0.001 |
| Thiamine (mg) | 1.6 ± 0.3 | 1.4 ± 0.3 | <0.001 |
| Riboflavin (mg) | 2.3 ± 0.5 | 2.1 ± 0.4 | <0.001 |
| Niacin (mg) | 27.7 ± 6.1 | 26.4 ± 6.0 | 0.028 |
| Niacin equivalents (mg) | 43.6 ± 7.8 | 41.3 ± 7.4 | 0.002 |
| Calcium (mg) | 1,114.5 ± 321.1 | 999.7 ± 263.3 | <0.001 |
| Iron (mg) | 13.1 ± 2.6 | 12.7 ± 2.7 | 0.14 |
| Sodium (mg) | 3,401.7 ± 558.4 | 3,188.6 ± 537.5 | <0.001 |
| Potassium (mg) | 3,406.4 ± 668.5 | 3,022.4 ± 559.6 | <0.001 |
| Magnesium (mg) | 408.8 ± 81.0 | 380.2 ± 74.4 | <0.001 |
| Zinc (mg) | 11.5 ± 1.6 | 11.2 ± 1.7 | 0.016 |
| Selenium (µg) | 142.6 ± 24.9 | 136.9 ± 25.7 | 0.016 |

^a^ Data are mean ± SD.

^b^ Adjusted for energy intake.

^c^ P was calculated according to the method of Benjamini and Hochberg.

EPA, eicosapentaenoic acid; DHA, docosahexaenoic acid; MUFA, monounsaturated fatty acids; PUFA, polyunsaturated fatty acids; SFA, saturated fatty acids; T1D, type 1 diabetes.
