# Supplementary material for: Improved adherence to Mediterranean Diet in adults with type 1 diabetes mellitus
Source: Eur J Nutr. 2018 Jul 17;58(6):2271–9. doi: 10.1007/s00394-018-1777-z (PMC6689285; doi:10.1007/s00394-018-1777-z)
Supplement: Supplementary file 3 — Supplementary material 3 (DOCX 18 KB) [file 394_2018_1777_MOESM3_ESM.docx]

Supplemental Table 2. Multivariate logistic regression for the Alternate Mediterranean Diet Score (aMED) and multivariate linear regression for the Alternate Healthy Eating Index (aHEI) of the study groups

| **Coefficients** | **aMED^a^** | | **aHEI^b^** | |
| --- | --- | --- | --- | --- |
|  | **OR (95% CI)** | **p** | **Estimate (95% CI)** | **p** |
| Intercept | 3.849 (0.551;26.882) | 0.17 | 30.580 (25.365;35.795) | <0.001 |
| T1D group | 0.528 (0.336;0.830) | 0.006 | 3.140 (1.955;4.326) | <0.001 |
| Physical activity | 0.599 (0.394;0.912) | 0.017 | 1.243 (0.104;2.381) | 0.033 |
| Age (years) | 0.970 (0.949;0.992) | 0.008 | 0.082 (0.024;0.141) | 0.006 |
| Male (sex) | 1.422 (0.936;2.160) | 0.09 | -1.853 (-2.952;-0.754) | 0.001 |
| Complete primary | 0.766 (0.258;2.277) | 0.63 | 1.919 (-0.931;4.769) | 0.19 |
| Secondary high cycle | 0.834 (0.285;2.439) | 0.74 | 2.360 (-0.488;5.207) | 0.10 |
| Graduate or higher | 0.401 (0.127;1.266) | 0.12 | 3.684 (0.687;6.679) | 0.016 |
| Smoker, current | 1.294 (0.806;2.079) | 0.29 | -0.492 (-1.785;0.800) | 0.46 |
| Smoker, former | 0.768 (0.450;1.311) | 0.33 | 0.924 (-0.438;2.286) | 0.18 |
| Site Lleida | 0.723 (0.471;1.109) | 0.14 | 2.557 (1.427;3.687) | <0.001 |
| BMI (kg/m^2^) | 0.999 (0.949;1.053) | 0.98 | -0.013 (-0.152;0.125) | 0.85 |
| Dyslipidemia | 1.286 (0.758;2.182) | 0.35 | -0.047 (-1.426;1.332) | 0.95 |
| Hypertension | 0.921 (0.471;1.800) | 0.81 | 0.477 (-1.198;2.153) | 0.58 |

^a^ Multivariate logistic regression for the alternate Mediterranean Diet Score (aMED) low group (0-2 points). Hosmer-Lemeshow test p-value: 0.47

^b^ Multivariate linear regression for the alternate Healthy Eating Index (aHEI). Multiple R^2^: 0.16; adjusted R^2^: 0.14.

BMI, body mass index; T1D, type 1 diabetes.
