# Supplementary material for: Improved adherence to Mediterranean Diet in adults with type 1 diabetes mellitus
Source: Eur J Nutr. 2018 Jul 17;58(6):2271–9. doi: 10.1007/s00394-018-1777-z (PMC6689285; doi:10.1007/s00394-018-1777-z)
Supplement: Supplementary file 4 — Supplementary material 4 (DOCX 17 KB) [file 394_2018_1777_MOESM4_ESM.docx]

Supplemental Table 3. Multivariate logistic regression for the Alternate Mediterranean Diet Score (aMED) and multivariate linear regression for the Alternate Healthy Eating Index (aHEI) of the type 1 diabetes group

| **Coefficients** | **aMED^a^** | | **aHEI^b^** | |
| --- | --- | --- | --- | --- |
|  | **OR (95% CI)** | **p** | **Estimate (95% CI)** | **p** |
| Intercept | 2.987 (0.199;44.649) | 0.43 | 30.999 (24.233;37.765) | <0.001 |
| Physical activity | 0.574 (0.292;1.125) | 0.11 | 1.662 (-0.038;3.363) | 0.05 |
| Male (sex) | 1.296 (0.683;2.459) | 0.43 | -2.099 (-3.641;-0.557) | 0.008 |
| Age (years) | 0.971 (0.936;1.008) | 0.13 | 0.121 (0.035;0.207) | 0.006 |
| Complete primary | 0.632 (0.156;2.548) | 0.52 | 1.594 (-1.802;4.989) | 0.36 |
| Secondary high cycle | 0.511 (0.128;2.038) | 0.34 | 2.369 (-1.089;5.827) | 0.18 |
| Graduate or higher | 0.220 (0.047;1.011) | 0.05 | 4.185 (0.539;7.831) | 0.025 |
| Smoker, current | 0.924 (0.431;1.981) | 0.84 | 0.157 (-1.678;1.993) | 0.87 |
| Smoker, former | 0.772 (0.336;1.774) | 0.54 | 1.915 (-0.016;3.846) | 0.05 |
| Site Lleida | 0.336 (0.172;0.659) | 0.001 | 3.956 (2.409;5.503) | <0.001 |
| BMI (kg/m^2^) | 1.019 (0.944;1.100) | 0.61 | -0.019 (-0.208;0.168) | 0.84 |
| Dyslipidemia | 1.227 (0.568;2.650) | 0.60 | 0.032 (-1.786;1.850) | 0.97 |
| Hypertension | 0.676 (0.278;1.640) | 0.39 | -0.168 (-2.184;1.848) | 0.87 |
| Diabetes duration (years) | 1.018 (0.981;1.056) | 0.35 | 0.032 (-0.051;0.114) | 0.45 |

^a^ Multivariate logistic regression for the alternate Mediterranean Diet Score (aMED) low group (0-2 points). Hosmer-Lemeshow test p-value: 0.16.

^b^ Multivariate linear regression for the alternate Healthy Eating Index (aHEI). Multiple R^2^: 0.20; adjusted R^2^: 0.15.

BMI, body mass index
